# *Bartonella henselae* Detected in Malignant Melanoma, a Preliminary Study

**DOI:** 10.3390/pathogens10030326

**Published:** 2021-03-10

**Authors:** Marna E. Ericson, Edward B. Breitschwerdt, Paul Reicherter, Cole Maxwell, Ricardo G. Maggi, Richard G. Melvin, Azar H. Maluki, Julie M. Bradley, Jennifer C. Miller, Glenn E. Simmons, Jamie Dencklau, Keaton Joppru, Jack Peterson, Will Bae, Janet Scanlon, Lynne T. Bemis

**Affiliations:** 1 T Lab Inc., 910 Clopper Road, Suite 220S, Gaithersburg, MD 20878, USA; mee@tlabdx.com; 2 Intracellular Pathogens Research Laboratory, Comparative Medicine Institute, College of Veterinary Medicine, North Carolina State University, Raleigh, NC 27607, USA; ebbreits@ncsu.edu (E.B.B.); rgmaggi@ncsu.edu (R.G.M.); julie_bradley@ncsu.edu (J.M.B.); 3 Dermatology Clinic, Truman Medical Center, University of Missouri, Kansas City, MO 64108, USA; drpderm@msn.com; 4 Department of Dermatology, University of Minnesota, Minneapolis, MN 55455, USA; maxwco@stanford.edu (C.M.); azarmaluki@gmail.com (A.H.M.); jamiedencklau@gmail.com (J.D.); Peteja@student.breckschool.org (J.P.); Baewi@student.breckschool.org (W.B.); Scanl113@umn.edu (J.S.); 5 Department of Biomedical Sciences, Duluth Campus, Medical School, University of Minnesota, Duluth, MN 55812, USA; melv0001@d.umn.edu (R.G.M.); gsimmons@d.umn.edu (G.E.S.J.); ktjoppru@gmail.com (K.J.); 6 Department of Dermatology, College of Medicine, University of Kufa, Kufa 54003, Iraq; 7 Galaxy Diagnostics Inc., Research Triangle Park, NC 27709, USA; jen.miller@galaxydx.com

**Keywords:** melanoma, A375 cells, *Bartonella* spp., *Bartonella henselae*, vector-borne pathogens, multi-immunostaining methods, confocal microscopy of thick sections, cancer microbiome, melanoma pathobiome

## Abstract

*Bartonella bacilliformis (B. bacilliformis)*, *Bartonella henselae (B. henselae)*, and *Bartonella quintana (B. quintana)* are bacteria known to cause verruga peruana or bacillary angiomatosis, vascular endothelial growth factor (VEGF)-dependent cutaneous lesions in humans. Given the bacteria’s association with the dermal niche and clinical suspicion of occult infection by a dermatologist, we determined if patients with melanoma had evidence of *Bartonella* spp. infection. Within a one-month period, eight patients previously diagnosed with melanoma volunteered to be tested for evidence of *Bartonella* spp. exposure/infection. Subsequently, confocal immunohistochemistry and PCR for *Bartonella* spp. were used to study melanoma tissues from two patients. Blood from seven of the eight patients was either seroreactive, PCR positive, or positive by both modalities for *Bartonella* spp. exposure. Subsequently, *Bartonella* organisms that co-localized with VEGFC immunoreactivity were visualized using multi-immunostaining confocal microscopy of thick skin sections from two patients. Using a co-culture model, *B. henselae* was observed to enter melanoma cell cytoplasm and resulted in increased vascular endothelial growth factor C (VEGFC) and interleukin 8 (IL-8) production. Findings from this small number of patients support the need for future investigations to determine the extent to which *Bartonella* spp. are a component of the melanoma pathobiome.

## 1. Introduction

Malignant melanoma is the sixth most common cancer reported by the Centers for Disease Control and Prevention (U.S. Cancer Statistics Working Group). Cutaneous melanoma maybe caused by a combination of sun exposure, genetic factors, and (as yet) unidentified environmental triggers [1]. An umbrella study of meta-analyses found that multiple phenotypic and environmental factors have been associated with the development of cutaneous melanoma, but collectively these studies suffer from heterogeneity and small study effects [2]. The meta-analyses indicate that, although genetic factors and UV irradiation are clearly part of the etiology of melanoma, there could be as yet unknown factors impacting the development of cutaneous melanoma [2].

Signatures of bacterial infection, including detection of DNA, RNA, and lipopolysaccharides, have been reported in many human solid tumors [3]. Recent studies indicate the association between bacteria and the tumor microbiome is complex and remains poorly understood [3,4,5]. Importantly, approximately 16% of human malignancies worldwide are linked to an infectious agent [6]. In the context of bacterial infections, previous studies have linked *Helicobacter pylori* to gastric cancer, *Chlamydia pneumoniae* with lung cancer, and *Salmonella typhi* with gallbladder cancer [7]. More recent studies suggest that *Streptococcus bovis* is associated with colorectal cancer [8,9]. With the advent of next generation sequencing (NGS) a tumor microenvironment pathobiome is being elucidated [10,11]. In the context of disease pathogenesis and potentially carcinogenesis, it is possible that specific microorganisms transition microbiomes to pathobiomes [11]. Recent studies in mice also suggest that the gut microbiome may influence the therapeutic response for cancers in locations distant from the gut [12,13]. Similarly, studies focusing on alterations of the gut microbiome in melanoma patients have documented variation in the melanoma treatment response to programed cell death protein 1 (PD-1) checkpoint inhibitors [14,15]. An early report about the microbiome on the outermost layer of the skin, the stratum corneum, by Salava et al., used noninvasive skin swabs of melanomas compared to the swab microbiome of benign nevi [16]. They reported minimal differences in the stratum corneum microbiome between cancer and benign nevi, although melanoma samples seemed to have slightly less microbiome diversity. In contrast, a study using a pig melanoma model found that microbiome diversity in full thickness biopsies from non-lesional skin had a significantly different microbiome from that observed in melanoma tissue [17]. That study examined a limited number of bacterial species and did not include *Bartonella.* Importantly, there are numerous defined mechanism by which bacteria could contribute to the development of cancer [18].

*Bartonella* spp. are emergent and neglected bacteria of worldwide distribution [19]. In humans, *Bartonella* spp. cause neurological symptoms, endocarditis, bacillary angiomatosis, bacillary peliosis, trench fever, and cat-scratch disease [20,21,22]. Bartonellosis is potentially fatal, especially in immunodeficient individuals [23]. Historically, persistent bloodstream infection has been documented in association with *B. bacilliformis* in patients with verruga peruana, after acquiring the infection via cutaneous sand fly bites in South America [24]. Persistent bloodstream infection with *B. henselae* has also been confirmed in association with immunocompromised cutaneous bacillary angiomatosis patients infected with HIV [25], potentially acquired by a cutaneous cat scratch or inoculation of flea feces subcutaneously. It is now increasingly clear that *Bartonella* spp. can induce chronic intravascular infections in humans, including symptomatic and healthy immunocompetent individuals [26,27,28]. In recent years, documentation of persistent bloodstream infection has been reported in “immunocompetent” blood donors and healthy veterinary workers [29,30]. What remains incompletely understood is the biological consequences of persistent bloodstream infection. Moreover, the extent to which long-standing dermal infection with these bacteria persists in the subcutaneous tissues of human beings is unknown. As stealth bacterial pathogens, a comparative medicine approach may facilitate enhanced understanding of the pathophysiology of Bartonellosis across animal species [20,29]. For example, bacillary angiomatosis, cutaneous panniculitis, and vasculitis involving the dermis occurs in both dogs and humans infected with *Bartonella* spp. [19,29,31]. Mechanistically, *Bartonella* spp. increase vascular endothelial growth factor C (VEGFC) secretion in association with cutaneous vasoproliferative tumor growth (bacillary angiomatosis, peliosis hepatis), which is also an important melanoma growth factor [18,32]. In this report, we describe *Bartonella* spp. exposure and infection in patients with cutaneous malignant melanoma. Our findings support the need for future studies to determine if *Bartonella* spp. are a prevalent component of the skin microbiome or if these bacteria contribute to the pathobiome leading to melanoma. 

## 2. Results

### 2.1. Demographics of Melanoma Patients

Demographic information, year of diagnosis, and patient reported co-morbidities for the eight malignant melanoma patients are included in Table 1. All participants volunteered for the study during August 2012 and were living in Oregon at the time of initial diagnosis; most had lived in Oregon for all (or a large portion) of their lives. Three had developed metastatic disease. All but one younger patient reported co-morbidities. 

### 2.2. Bartonella spp. Seroreactivity, Enrichment Blood Culture, and PCR

*Bartonella* spp. serology and *Bartonella Alphaproteobacteria* growth medium (BAPGM) enrichment blood culture PCR results are summarized in Table 2. In 2012, at North Carolina State University, *B. henselae* DNA was amplified and sequenced from blood or from BAPGM enrichment blood cultures from 3 of 8 melanoma patients (patients 2, 4, and 7). All patient DNA was subjected to both qPCR and conventional PCR (cPCR). Five patients were *Bartonella* spp. seroreactive, only one of whom was PCR/DNA sequence positive for *B. henselae*. The two remaining *B. henselae* PCR positive patients were immunofluorescence antibody assay (IFA) non-seroreactive at all screening dilutions to all four *Bartonella* spp. test antigens. 

Previously, collected formalin-fixed paraffin embedded (FFPE) melanoma biopsy tissue from patients 4 and 7 were retrievable for hemi-nested PCR conducted at the University of Minnesota. *B. henselae* DNA was PCR amplified and sequenced from FFPE melanoma tissue blocks from patients 4 and 7.

### 2.3. Confocal Immunohistochemistry of Melanoma Tissues

Tissue sections from two patients, 4 and 7, FFPE tissue blocks were immunostained and examined using previously validated imaging protocols [33,34]. Sections were multi-stained with a vascular biomarker anti-collagen-type 4, a lymphatic vessel biomarker (anti-LYVE1), VEGFC, anti-B. henselae, and a DNA stain for tissue localization. The dermal-epidermal boundary was disrupted as revealed by a diffuse staining pattern of collagen-type 4 immunostaining (data not shown).

**Figure 1:**
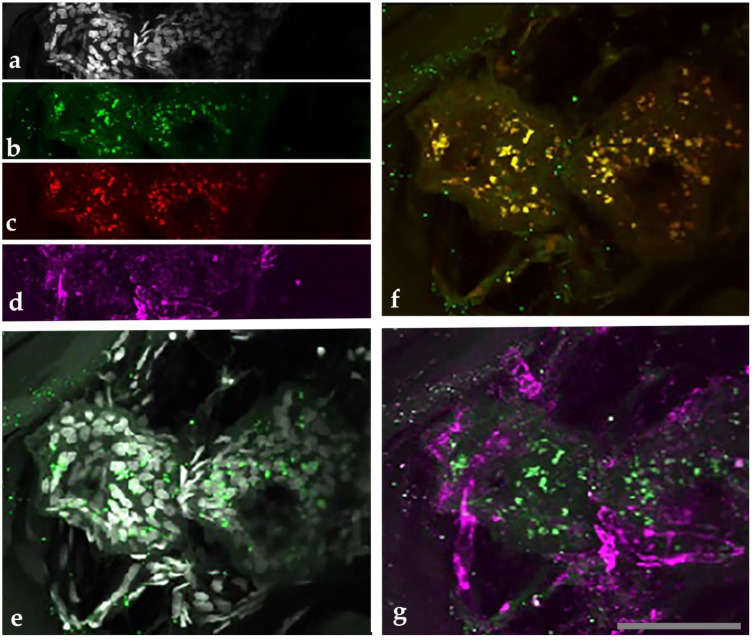
*Bartonella henselae* was detected in a melanoma biopsy, multi-stained with antibodies to *Bartonella henselae*, vascular endothelial growth factor C (VEGFC), lymphatic vessel biomarker (LYVE1), and DAPI nuclear dye. A thick tissue section, 80 microns, was multi-stained with (**a**) nuclear stain DAPI (white), (**b**) *B. henselae* antibodies (green), (**c**) VEGFC antibodies (red), and (**d**) lymphatic vessel biomarker LYVE1 antibodies (magenta) and imaged with laser scanning confocal microscopy. Merged channels, (panels (**e**–**g**)) reveal that immunoreactive-*B. henselae* (green in (**b**,**e**–**g**)) co-localizes with the nuclear dye DAPI (white in **a**,**e**), colocalizes with immunoreactive VEGFC (red in (**c**,**f**)) and appears to be inside the lymphatic vessels (magenta in (**d**,**g**)). Sequential tissue sections that received no *B. henselae* antibodies show no contribution to the fluorescence signal (data not shown). The 40X UPLFLN NA:1.3, 33 microns total Z-stack thickness, 61 steps, 0.55 micron steps. Scale bar = 50 microns.

By immunostaining, *B. henselae* and VEGF co-localized in the melanoma tissues from patients 4 and 7 (Figure 1). Merged images of *B. henselae* immunoreactivity revealed a close association of the bacteria with pro-angiogenic VEGFC within dermal lymphatic vessels (Figure 1, merged). *Bartonella henselae-*immunoreactivity was not visualized in the epidermis. 

In general, *Bartonella* spp. immunoreactivity was distributed in close association with the melanoma cells and dermal lymphatics. There was no endogenous tissue fluorescence using no-primary-no-secondary-antibody controls; and no non-specific binding was detected when only the secondary antibody was used in the staining protocol (controls are included in Figure S1). 

### 2.4. Co-Culture of B. henselae with Melanoma Cells (A375)

**Figure 2:**
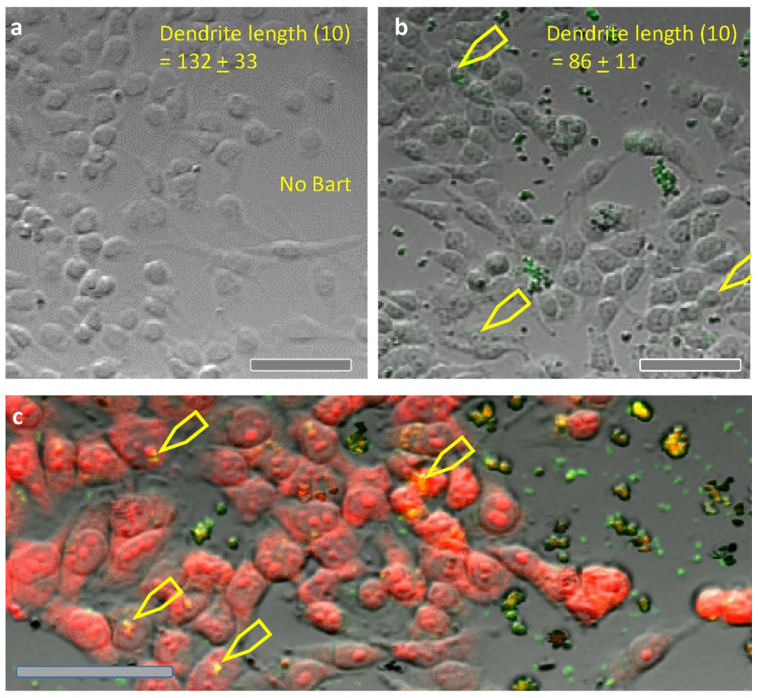
Melanoma A375 cells co-cultured with *B. henselae*. (**a**) Melanoma A375 cells were cultured without and (**b**) with *B. henselae* organisms (green). After 24 h, cultured cells were fixed and immunostained with anti-*Bartonella* spp. (green). The length of dendritic processes of the Melanoma A375 cells were shorter when cultured with *B. henselae* (t_2_ = 4.18, *p* < 0.001 (*n* = 10). (**c**) At 24 h, immuno-reactive *B. henselae* (green) appeared to be within as well as outside of melanoma A375 cells. Z-projection of merged *Bartonella* spp.-immunoreactivity (green), DNA stain TOPRO3 (red), and differential interference contrast (DIC) imaging. Single optical sections were captured with confocal microscopy at 10× UPLSAPO NA: 0.40. Scale bar = 50 microns.

*Bartonella henselae* is an intracellular bacterium that induces expression of VEGFC [35,36,37]. Additionally, *Bartonella* spp. are known to cause vasoproliferative lesions in the human host [38]. A co-culture model was developed to determine if *B. henselae* altered the biological behavior of cultured melanoma cells (Figure 2b). Melanoma cells inoculated with a live culture of *B. henselae* American Type Culture Collection (ATCC), # 49882, Houston-1), had shorter dendritic processes as compared to non-infected melanoma cells (Figure 2a). 

**Figure 3:**
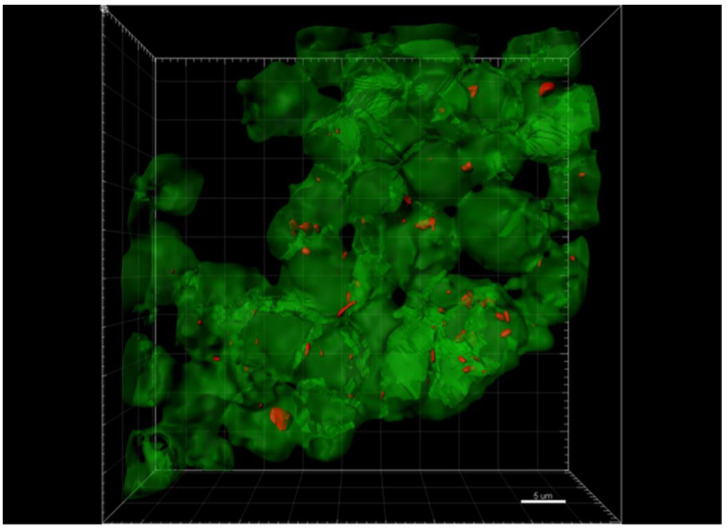
A video documenting the intracellular location of *B. henselae* in melanoma cells. Melanoma A375 cells were cultured with *B. henselae* organisms and at 24 h co-cultures were stained with the nuclear dye Syto9. Z-projection total thickness is 19.3-microns, constructed from 64 optical sections each 0.3 microns thick (Nikon Ti-E Motorized Microscope. Plan Fluor 100× Oil DIC H, Nikon. Scale bar = 5 microns). Data were deconvoluted using Imaris microscopy software with a 5-micron size filter was applied to detect bacteria and only bacteria within the melanoma cells are shown. AVI movie file of this Z-stack is shown in the Video S1 movie file.

In a first step to determine if the bacteria are in melanoma cells, a three-dimensional (3D)-video of the co-culture was generated (Figure 3, Supplementary Materials).

### 2.5. Cytokine Analysis of Melanoma A375 Cells Co-Infected with B. henselae

**Figure 4:**
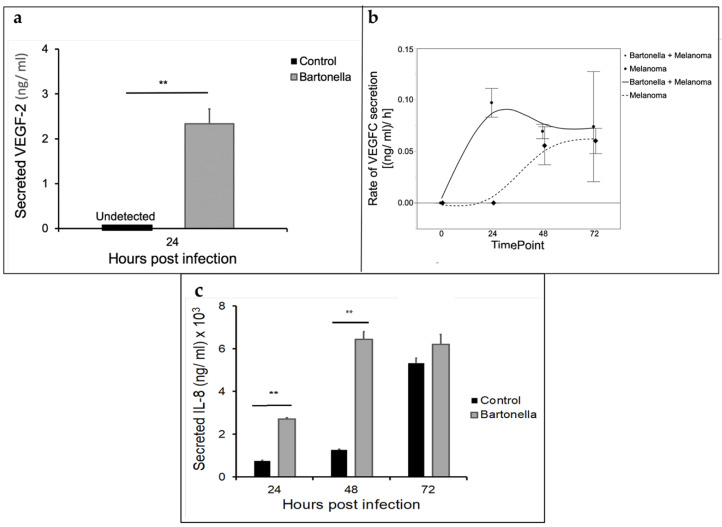
*Bartonella henselae* triggers cytokine release during co-culture with melanoma cells. (**a**) An ELISA documented a significant increase in VEGFC expression at 24 h** after inoculation of melanoma cells with *B. henselae* (Student’s *t*-test: t_2_ = 7, *p* < 0.01, *n* = 6). Significance was determined by Student’s *t*-test. (**b**) VEGFC expression was greater in co-cultures and the increase in VEGFC-expression occurred earlier, namely 24 h, when *B. henselae* was present as compared to melanoma cells alone. The rate of VEGFC expression when *B. henselae* was present was significantly greater than that for melanoma cells alone at 24 h (t_4_ = 7, *p* < 0.05), but did not differ from that of melanoma alone after 48 (t_4_ = 0.71, *p* = 0.54) and 72 h (t_4_ = 0.25, *p* = 0.82). *n* = 6. (**c**) After inoculation of A375 melanoma cells with *B. henselae*, media was collected and analyzed for interleukin 8 (IL-8) expression at 24, 48, and 72 h. IL-8 expression was significantly increased in response to the presence of Bartonella at 24 and 48 h time points ** (Student’s *t*-tests: t_4_ = 27.08, *p* < 0.001, and t_4_ = 14.84, *p* < 0.001 for 24 and 48 h, respectively). IL-8 expression was higher in the presence of Bartonella at 72 h, but the difference was not significant (t_4_ = 1.72, *p* = 0.16). *n* = 6.

*Bartonella henselae* co-cultured with melanoma cells induced production of VEGFC and interleukin 8 (IL-8) (Figure 4). VEGFC was significantly upregulated at 24 h in *B. henselae-*infected melanoma cells as compared to melanoma cells alone (Figure 4a). IL-8 cytokine expression was significantly increased at 24 h of incubation in *B. henselae* co-cultures (Figure 4c).

## 3. Discussion

This study provides, serological and enrichment blood culture/PCR evidence that a subset of melanoma patients have been exposed to or are infected with *B. henselae.* Importantly, *B. henselae* DNA was amplified and sequenced from enrichment blood cultures from patients 4 and 7 at North Carolina State University, and again years later from FFPE melanoma tissues at the University of Minnesota. For both of these patients, there was a 4-year time span between the initial tissue biopsy and the documentation of blood borne *B. henselae* infection.

*Bartonella henselae* DNA was amplified and sequenced from the blood or BAPGM enrichment blood cultures of three patients, two of whom were not seroreactive to any of the four *Bartonella* spp. IFA antigens. Previous studies have confirmed variation in the sensitivity of IFA assays depending upon the strain type used as antigen [30,37]. In addition, and for reasons that lack clarity, a subset of patients with persistent *Bartonella* spp. bloodstream infections can be seronegative to the infecting species and strain used for IFA testing [39,40]. Whether this is a laboratory phenomenon (lack of IFA-assay sensitivity) or a patient phenomenon (anergy) remains unclear.

It is well documented that *Bartonella* spp. can be difficult to detect by standard microscopic histopathological techniques [41]. In this study of two FFPE melanoma tissues, confocal immunohistochemistry supported the localization of *Bartonella* spp. in the dermal lymphatics. Interestingly, Hong and colleagues using an experimental rat infection study found that *B. tribocorum* first invades the cutaneous lymphatics, prior to establishing intraerythrocytic and endothelial cell infections [42]. In melanoma tissue, immunoreactive-*Bartonella* spp. appeared to co-localize with VEGFC within the lymphatic vessels. Interestingly, in addition to promoting endothelial cell proliferation, VEGFC plays a dominant role in lymphatic vessel growth [43]. Uniquely several *Bartonella* spp., including *B. henselae*, stimulate VEGF production resulting in the proliferation of blood vessels [44,45], which is also a hallmark of malignant melanoma [46]. In vitro infection of melanoma cell cultures with *B. henselae* also resulted in changes in cell morphology (Figure 2). The mechanism of bacterial entry into the melanoma cells, as compared to invasome-mediated entry of *B. henselae* into endothelial cells [47], deserves future study. 

*Bartonella* spp. infection is associated with increased pro-angiogenic cytokine expression as well as inhibition of apoptosis in endothelial cells [48,49]. The finding that VEGFC is upregulated in melanoma cells co-cultured with *B. henselae* is similar to previously reported studies of *B. henselae* co-cultures with endothelial or HeLa cells [47,49,50]. *Bartonella henselae* has also been shown to increase IL-8 expression in endothelial cell lines [51]. Similarly, IL-8 levels increased in *B. henselae-*inoculated melanoma cell media compared to melanoma cells alone.

The initial 2012 microbiological results reported in this study were remarkable and unexpected. Subsequent collaborative efforts with confocal imaging and the development of the melanoma/*B. henselae* co-culture model, which spanned several years, further supported a potential role for Bartonella infection in the pathogenesis of melanoma in a subset of patients. There are several limitations associated with this study. As the eight study participants were from the same geographic location and all were tested in the same month of August, these results cannot be extrapolated to other melanoma patients or other geographic locations. As all patients were diagnosed with melanoma years before study entry, and because recent studies have found *Bartonella* spp. bloodstream infections in healthy individuals [30], the medical relevance of the association of this genus of bacteria with melanoma awaits future studies. In addition to the small number of study participants, only two FFPE tissue blocks were retrievable, limiting documentation of tumor-associated *Bartonella* spp. DNA, as well as potential visualization of bacteria within the tumor. Only a limited number of cytokine studies were performed with the newly developed *B. henselae*/co-culture model, clearly an opportunity for future utilization of this cell culture infection model. 

In conclusion, the results generated in this study may prove to be of potential importance to future investigations of melanoma carcinogenesis. It is possible that vector transmitted *Bartonella* spp. are a potential component of a microbial pathobiome in a subset of melanoma patients. The *B. henselae*-A375 melanoma co-culture model can be used in future studies to characterize impacts of Bartonella infection on melanoma pathogenesis. Most importantly, this initial observational study provides the background and justification for future funded studies, addressing the impact of *Bartonella* spp. as part of the cutaneous microbiome or melanoma pathobiome.

## 4. Materials and Methods

### 4.1. Study Timeline

Given the documented association of *Bartonella* spp. with cutaneous vasoproliferative lesions, panniculitis and rash, and based upon his clinical suspicion, Dr. Paul Reicherter (dermatologist) contacted Dr. Breitschwerdt (infectious disease researcher) to pose the question: “Could *Bartonella* spp. infection be involved in the pathogenesis of cutaneous melanoma in people with minimal exposure to sunlight?” To initially address this question, blood from eight patients with a previous diagnosis of malignant melanoma were tested for serological or enrichment blood culture/PCR evidence of *Bartonella* spp. exposure/infection. Subsequently, the formalin-fixed, paraffin-embedded (FFPE) biopsy specimens from two of these patients were retrievable for molecular testing and attempted visualization of *Bartonella* spp. in their melanomas. Later, in an attempt to better understand our initial patient-oriented observations, a co-culture model using A375 melanoma cells inoculated with *B. henselae* was developed. The co-culture model facilitated visual localization of the bacteria and the use of assays to determine if the *B. henselae* influenced the cytokine expression of the cultured melanoma cells. For various reasons, these microbiological, imaging, and co-culture studies spanned a 7-year period from the time of blood collection and as long as 6 years from the time of initial melanoma diagnosis to initial microbiological testing.

### 4.2. Patients and Samples

During the month of August 2012, all patients previously diagnosed with melanoma that were being followed by a Dermatology Clinic in Oregon were offered the opportunity to participate in the study. Patients were included regardless of melanoma location, the time since initial diagnosis, and whether there had been reoccurrence or metastasis. Eight individuals agreed to participate in the study. Institutional Review Board approval (North Carolina State University Institutional Review Board (IRB) #1960, Detection of *Bartonella* Species Infection among a Cohort of Healthy and Sick People) was obtained for the study and each individual provided a signed consent for testing and study participation. Dr. Reicherter submitted blood and serum samples from these eight melanoma patients to the Intracellular Pathogens Research Laboratory, Comparative Medicine Institute, College of Veterinary Medicine, North Carolina State University for *Bartonella* spp. serology and enrichment blood culture/PCR testing.

*Bartonella* spp. immunofluorescence antibody assay (IFA) serology (four independent IFA assays) was performed as previously described [39]. Cell culture grown *Bartonella vinsonii* subsp. *berkhoffii, Bartonella henselae-*Houston I and -San Antonio 2 strains and *Bartonella koehlerae* were used as antigens. Sera were screened at dilutions of 1:16, 1:32, and 1:64 and endpoint titer. Antibody titers of 1:64 or greater were considered seroreactive.

Aseptically obtained whole blood and serum specimens were tested by PCR and following *Bartonella* alpha-Proteobacteria growth medium (BAPGM) enrichment blood culture, as previously described [40,52]. The BAPGM platform incorporates six separate PCR tests, each representing a different component of the testing process for each patient sample: (1) and (2) PCR amplification of *Bartonella* spp. following DNA extraction from whole blood and from serum; (3)–(5) PCR following BAPGM enrichment of whole blood culture incubated for 7, 14, and 21 days; and (6) PCR from subculture isolates if obtained after sub inoculation from the BAPGM flask onto plates containing trypticase soy agar with 5% sheep blood that are incubated for 4 weeks. PCR specimen preparation, DNA extraction, and PCR amplification and analysis were performed in three separate rooms with unidirectional workflow to avoid DNA contamination. In addition, BAPGM cultures were processed in a biosafety cabinet with Hepa filtration in a limited access Biosafety Level III laboratory. PCR-negative controls were prepared using 5 μL of DNA from the blood of a healthy dog, and *B. henselae* (Houston 1 strain) at a concentration of 1 genome copy/ μL was used as a PCR-positive control during the entire course of this study. In no instance was *B. henselae* or DNA of any other *Bartonella* sp. amplified in the negative control lane on any PCR gel. To assess for potential contamination during blood sample processing into BAPGM, a non-inoculated BAPGM culture flask was processed simultaneously and in an identical manner with each batch of patient blood and serum samples tested. For all components of the BAPGM platform (PCR from blood, serum, enrichment cultures at 7, 14, and 21 days, and subcultures), PCR-negative controls remained negative throughout the course of the study. In addition, subcultures of non-inoculated BAPGM medium (culture control) at 7, 14, and 21 days did not yield bacterial growth. 

### 4.3. Conventional PCR (PCR) and Quantitative PCR (qPCR) Analysis

Conventional PCR screening of *Bartonella* ITS region was performed using oligonucleotides 325s: 5′ CTT CAG ATG ATG ATC CCA AGC CTT CTG GCG 3′ and 1100as: 5′ GAA CCG ACG ACC CCC TGC TTG CAA AGC A 3′ as forward and reverse primers, respectively. Amplification was performed in a 25-µL final volume reaction containing 12.5 µL of MyTaq Premix (Bioline, Memphis, TN, USA, 0.2 µL of 100 µM of each forward and reverse primer (IDT® DNA Technology, Coralville, IA, USA), 7.5 µL of molecular–grade water, and 5 µL of DNA from each sample tested. Conventional PCR was performed in an Eppendorf Mastercycler EP Gradient® under the following conditions: a single hot-start cycle at 95 °C for 2 min followed by 55 cycles of denaturing at 94 °C for 15 s, annealing at 68 °C for 15 s, and extension at 72 °C for 18 s. Amplification is completed by an additional cycle at 72 °C for 1 min, and products were analyzed by 2% agarose gel electrophoresis with detection using ethidium bromide under ultraviolet light. Amplicons products were sequenced to establish species strain identification. Quantitative PCR screening of the *Bartonella* ITS region was performed using oligonucleotides 325s: 5′ CTTCAGATGATGATCCCAAGCCTTCTGGCG 3′ and 543as: 5′ AATTGGTGGGCCTGGGAGGACTTG 3′ as forward and reverse primers, respectively, and oligonucleotide BsppITS438probe: 5′ FAM-GGTTTTCCGGTTTATCCCGGAGGGC-BHQ1 3′ as TaqMan probe. Amplification is performed in a 25-µL final volume reaction containing 12.5 µL of SsoAdvanced™ Universal Probes Supermix (Bio-Rad, Hercules, CA, USA), 0.2 µL of 100 µM of each forward primer, reverse primer, and TaqMan probe (IDT® DNA Technology, Coralville, IA, USA), 7.5 µL of molecular-grade water, and 5 µL of DNA from each sample tested. Quantitative PCR was performed in an CFX96® (Bio-Rad) under the following conditions: a single hot-start cycle at 95 °C for 3 min followed by 45 cycles of denaturing at 94 °C for 10 s, annealing at 68 °C for 10 s, and extension at 72 °C for 10 s. Amplification was completed by an additional cycle at 72 °C for 30 s. PCR negative controls were prepared using 5 µL of DNA from blood of a healthy dog. Positive controls for PCR were prepared by using 5 µL of DNA from a serial dilution (using dog blood DNA) of *B. henselae* genomic DNA equivalent to 0.1, 0.01, and 0.001 pg/µL. Positive amplification was assessed by analysis of detectable fluorescence vs. cycle values for qPCR. Positive amplicons products from either conventional or qPCR were sent for sequencing to establish species strain identification.

### 4.4. PCR Analysis of DNA Extracted from Formalin-Fixed Tissue

A 2mm cube of tissue was excised from each paraffin block with a new sterile blade and deparaffinized using two changes of xylene heated for 3 min to 56 °C between changes. Once the tissue was deparaffinized, DNA was extracted following the methods described for the AllPrep RNA and DNA Tissue kit from Qiagen (Valencia, CA, USA). DNA was amplified using a Hemi-Nested PCR protocol for *Bartonella* spp. The first round of PCR used previously published primers designed to amplify a region conserved in multiple species of *Bartonella* [53]. These primers amplify sequences in the 16S-23S ribosomal intragenic region (Bspp 325s and 1100as). A second reverse primer was designed to amplify a smaller region in the original amplicon, still using the previously published forward primer (Bspp 325s) and the new reverse primer (5’- GAG GAC TTG AAC CTC CGA CCT CAC G). Each PCR run started with touch down PCR (with half degree step down from 65 °C to 57 °C) followed by 40 cycles at 60 °C and a final extension at 72 °C for five min. GoTaq Green Polymerase (Promega, Fitchburg, WI, USA) was used for all PCR amplifications. The amplified PCR products were then gel extracted and cloned into T-vector cloning vector pCR2.1 TOPO provided in the TOPO cloning Kit (product number K4560-01, Invitrogen by Life Technologies, Waltham, MA, USA). Plasmids were subjected to direct sequencing following submission to the University of Minnesota Genomics Center (UMGC). Sequences were compared to reported sequences for *Bartonella* species using the Blast program. 

### 4.5. Multi-Staining and Confocal Imaging of Tissues and Co-Cultures

Initially, approximately 2 × 2 × 2 mm blocks of lesional skin tissue were excised from the FFPE blocks with a new scalpel blade and the excised block pieces were deparaffinized [33]. The tissue was mounted in optimal cutting temperature compound (OCT) and vertical sections, 80-microns thick, were cut on a cryostat. Sections were floated in glass dishes for incubations with blocking serum, primary and secondary antibodies following previously developed protocols [33,34,54]. Sections were multistained with combinations of the following primary and secondary antibodies and nuclear dyes. Figure 1: nuclear dye DAPI, anti-*Bartonella henselae* made in mouse at 1:100 (AB704, Abcam Cambridge, MA, USA) plus anti-mouse IgG Alexa 488 made in donkey at 1:400 dilution (Jackson ImmunoResearch), anti-VEGFC made in goat at 1:200 (AB18883 Abcam, Cambridge, MA, USA), plus anti-goat IgG made in donkey conjugated to Alexa 568 at 1:800 (Jackson ImmunoResearch), and anti-LYVE1 made in rabbit at 1:400 (485214 US Biological, Salem, MA, USA), plus anti-rabbit IgG made in donkey conjugated to Alexa 647 at 1:800. Figure 2 and Figure S1: anti-*Bartonella* spp. made in chicken at 1:100 dilution (produced by Aves Labs, Inc., Davis, CA, USA), plus anti-chicken IgY conjugated to Alexa 568 at 1:400 dilution (Jackson ImmunoResearch), and nuclear dye TOPRO3 at 1:5000 (T3605, Thermo Fisher, Waltham, MA, USA). The anti-*Bartonella* spp. antibody recognized the gltA citrate synthase region, conserved in all *Bartonella* spp. The nuclear dye Syto9 at 20 micromolar (L-7007, Thermo Fisher, Waltham, MA, USA) was used in Figure 3. The protocol for titration of the new *Bartonella* spp. antibody made in chickens is described in the supporting information Figure S1. Cultures were fixed in formalin for 30 min and stained within the plate wells over 2 days. Confocal microscopy was used to image multistained tissues and cells. 

### 4.6. Co-Culture of Melanoma Cells and B. henselae

The melanoma cell line A375 was obtained from the ATCC (# CRL-1619) and maintained in RPMI media supplemented with 10% fetal bovine serum in a humidified incubator with 5% CO_2_ at 37 °C. The green fluorescent protein (GFP) expressing plasmid pEGFP-C1 was transfected into A375 melanoma cells and selected using neomycin for a stable GFP expressing clone as previously described [55]. The melanoma cell line, A375, was grown to confluence and then replated at a concentration of 1 × 10^4^ cells/ml, after which the bacteria were directly added to the cell culture media of the A375 cells. The bacteria were added at a concentration of 5 × 10^5^ cells per well. Media from triplicate wells of co-culture was collected at 24, 48, and 72 h post inoculation for cytokine analyses. For imaging, the A375-GFP cells were grown to confluence and then one plate was inoculated with *B. henselae* (ATCC, # 49882, Houston-1). The cells were fixed after 72 h of growth in the presence of *B. henselae* with 10% formalin for 30 min and then transferred to PBS. Then formalin fixed cells were immunostained with an antibody targeting *Bartonella* spp. and nuclear dye TOPRO3 or Syto9 and imaged by confocal microscopy. The cell line A375 was authenticated by IDEXX BioAnalytics (Columbia, MO, USA) using short tandem repeat (STR) analysis in February 2020. 

### 4.7. Cytokine Analysis

Samples of culture media supernatants collected at 24, 48, and 72 h from A375 cells inoculated with or without *B. henselae* were submitted for testing at the Cytokine Reference Laboratory (CRL, University of Minnesota, Minneapolis, MN, USA). This is a Clinical Laboratory Improvement Amendments of 1988 licensed facility. Samples were analyzed for VEGF and IL-8, and tumor necrosis factor alpha (TNF-alpha) using the Luminex platform with multiplexing. The magnetic bead set was purchased from R&D Systems, Minneapolis, MN, USA. Samples were assayed according to manufacturer’s instructions. Fluorescent color-coded beads coated with a specific capture antibody were added to each sample. After incubation and washing, biotinylated detection antibody was added followed by phycoerythrin-conjugated streptavidin. The beads were read on a Luminex instrument (BioPlex 200, Hercules, CA, USA), which is a dual-laser fluidics-based instrument. One laser determines the analyte being detected via the color coding; the other measures the magnitude of the PE signal from the detection antibody, which is proportional to the amount of analyte bound to the bead. Samples from all testing time points were run in duplicate for each of the assays. Values were interpolated using 5-parameter fitted standard curves.

### 4.8. Statistical Methods

Student’s *t*-tests were performed using JMP statistical software (SAS Institute, Cary, NC, USA). *p*-values were corrected to control the experiment-wise error rate using the Dunn-Sidak procedure [56].

### 4.9. Ethics Approval and Consent to Participate

All procedures performed in studies involving human participants were in accordance with the ethical standards of the institutions and/or national research committee and with the 1964 Helsinki declaration and its later amendments or comparable ethical standards. North Carolina State University (NCSU) Institutional Review Board (IRB 1960) provided ethical approval for this study. Written informed consent was obtained from the participants included in this study. The Institutional Review Board from North Carolina State University (IRB #1960) approved a research study investigating the detection of *Bartonella* species infection among a cohort of healthy and sick people. Dr. Reicherter submitted blood and serum samples from eight melanoma patients who signed written informed consent to the Intracellular Pathogens Research Laboratory, Comparative Medicine Institute, College of Veterinary Medicine, North Carolina State University.

## Figures and Tables

**Table 1:** Demographic data for eight melanoma patients from Oregon.

Patient No:	Age (Years)	Gender	Occupation	Oregon Residency (Years)	Year of Melanoma Diagnosis	Patient Reported Co-Morbidities *
**1**	81	F	Housewife	64	29 December 2011 Malignant melanoma (superficial spreading type)	Breast cancer, dementia, cholecystectomy, hysterectomy, arteriosclerosis
**2**	77	M	Dentist	77	2008 Metastatic	Hairy cell leukemia, lung cancer, depression, diabetes, hypertension, hypothyroid, chronic pain
**3**	71	M	Truck driver/boiler operator	71	23 August 2006 Malignant melanoma (superficial spreading type)	Depression, diabetes, 2 heart attacks, hypertension, hypercholesterolemia
**4**	68	M	Painter/truckdriver	5	Primary 2008 ** Metastatic 2009 Malignant melanoma (invasive)	Thrombosis, heart bypass, heart attack, diabetes, hypertension, hypercholesterolemia
**5**	71	M	Physician	39	17 March 2010 Malignant melanoma (in situ lentigo malignant type)	Carpel tunnel syndrome, acromioplasty, basal cell/squamous cell, hypertension, hypercholesterolemia
**6**	85	F	Nurse	80	Primary 15 August 2006 Malignant melanoma (nodular) Recurrence 2011 and 2012	Breast Cancer, depression, hysterectomy, diabetes, replaced heart valve (bovine), hypertension, hypercholesterolemia, cholelithiasis
**7**	31	M	Cook	14	First lesion 2 January 2008 Malignant melanoma (invasive) Second lesion 16 February 2012 ** Malignant melanoma (invasive) Third lesion 7 February 2018 Malignant melanoma (superficial spreading type)	Appendectomy
**8**	77	M	Timber worker	42	Primary 17 December 2007 Malignant melanoma (superficial spreading type) Second lesion 3 January 2008 Malignant melanoma	Diabetes, Hypertension, Hypercholesterolemia, Asthma

* Co-morbidities were self-reported on the Institutional Review Board (IRB) approved questionnaire completed by each participant. ** Tissue blocks were obtained from these dates.

**Table 2:** *Bartonella* spp. serology and *Bartonella* alpha proteobacteria enrichment blood culture PCR results for eight melanoma patients from Oregon.

Reciprocal IFA Antibody Titers To:					
**Patient No:**	***Bartonella vinsonii berkhoffii***	***Bartonella henselae* Houston 1**	***Bartonella henselae* San Antonio 2**	***Bartonella koehlerae***	*Bartonella Alphaproteobacteria* Growth Medium (BAPGM) Enrichment Blood Culture/PCR *
**1**	**64**	<16	**64**	<16	Negative
**2**	**64**	<16	**128**	<16	***B. henselae (qPCR)***
**3**	**128**	<16	32	<16	Negative
**4**	<16	<16	<16	<16	***B. henselae (cPCR)***
**5**	<16	<16	<16	<16	Negative
**6**	<16	<16	**64**	<16	Negative
**7**	<16	<16	<16	<16	***B. henselae (cPCR)***
**8**	**64**	16	**64**	**64**	Negative

* Bartonella species confirmed by DNA sequence analyses were at least 99% similar to *B. henselae*.

## Data Availability

Data is contained within the article or supplementary material The data presented in this study are available in [*Bartonella henselae* Detected in Malignant Melanoma, a Preliminary Study].

## Supplementary Materials

The following are available online at https://www.mdpi.com/2076-0817/10/3/326/s1, Figure S1: characterization of *anti-Bartonella* spp. IgY. A new IgY antibody to *Bartonella* spp. was generated in chickens (Aves Lab, Inc., Davis, CA, USA). Optimal dilutions and the ability of this new IgY antibody to identify two species of *Bartonella* are presented. Video S1: confirmation of *Bartonella henselae* invasion of melanoma A375 cells. AVI movie file of this Z-stack.
