# Supplementary material for: Bartonella henselae Detected in Malignant Melanoma, a Preliminary Study
Source: Pathogens. 2021 Mar 10;10(3):326. doi: 10.3390/pathogens10030326 (PMC7998106; doi:10.3390/pathogens10030326)
Supplement: Supplementary file 1 [file pathogens-10-00326-s001.zip › S1_Fig.pdf]

Chicken Primary Antibody Titration Images. The following images were gathered from a titration experiment on IgY chicken primary antibody using a 100x objective on a fluorescent microscope. Cultures of *Bartonella henselae* and *Bartonella tamiae* were incubated with different concentrations of anti-*Bartonella* spp. IgY and different concentrations of the secondary reporter antibody, anti-chicken conjugated to Alexa 568. The concentrations of the chicken primary antibody were 1/100, 1/500, and the secondary antibody was also used at 1/100, 1/500, and 1/2000. The same procedure was duplicated with anti-chicken conjugated to Alexa 647 with comparable results. Finally, control sections containing secondary antibodies only, or no primary or secondary antibodies and one containing neither secondaries nor primaries revealed no fluorescence signal;. DAPI was used to visualize the bacteria. Scale bar = 100 microns.

IR: Immunoreactivity
B. h.: *Bartonella henselae*
B. t.: *Bartonella tamiae*
1°: concentration of primary antibody- chicken *α Bartonella spp.* IgY
2°: concentration of secondary antibody- goat *α* chicken Alexa Fluor 568

Images of Bartonella henselae and tamiae bacteria using a 100x objective on a fluorescent microscope			
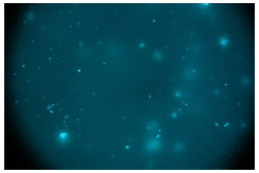<br>B. h. - IR<br>1/100 IgY<br>1/100 α ch - A568<br>(UV filter)	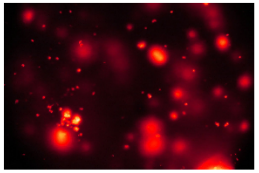<br>B. h. - IR<br>1/100 IgY<br>1/100 α ch - A568<br>(re d filter)	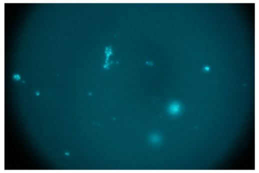<br>B. h. - IR<br>1/100 IgY<br>1/500 α ch - A568<br>(UV filter)	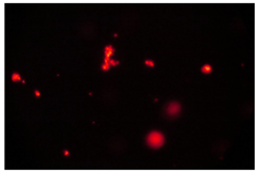<br>B. h. - IR<br>1/100 IgY<br>1/500 α ch - A568<br>(red filter)
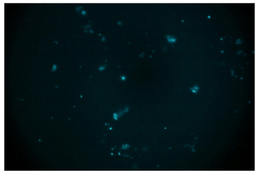<br>B. t. - IR<br>1/100 IgY<br>1/100 α ch - A568<br>(UV filter)	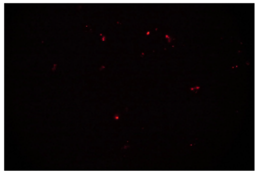<br>B. t. - IR<br>1/100 IgY<br>1/100 α ch - A568<br>(red filter)	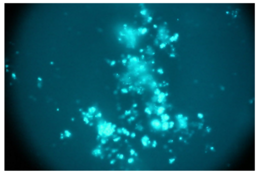<br>B. t. - IR<br>1/500 IgY<br>1/100 α ch - A568<br>(UV filter)	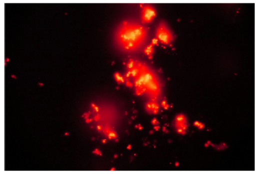<br>B. t. - IR<br>1/500 IgY<br>1/100 α ch - A568<br>(red filter)
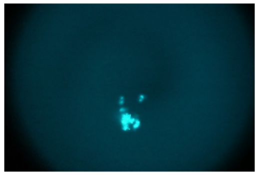<br>B. h. - IR<br>0 IgY<br>0 α ch - A568<br>(UV filter)	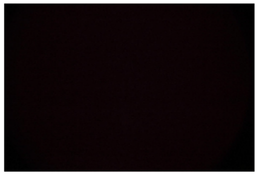<br>B. h. - IR<br>0 IgY<br>0 α ch - A568<br>(red filter)	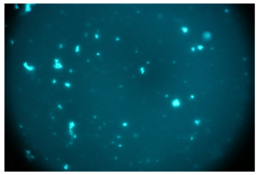<br>B. t. - IR<br>0 IgY<br>0 α ch - A568<br>(UV filter)	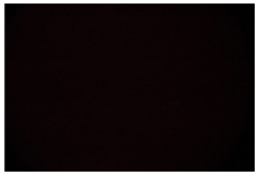<br>B. t. - IR<br>0 IgY<br>0 α ch - A568<br>(red filter)
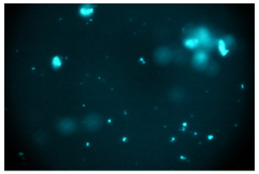<br>B. t. - IR<br>0 IgY<br>1/100 α ch - A568<br>(UV filter)	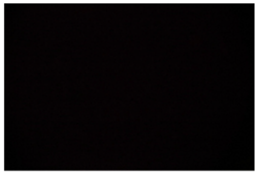<br>B. t. - IR<br>0 IgY<br>1/100 α ch - A568<br>(red filter)	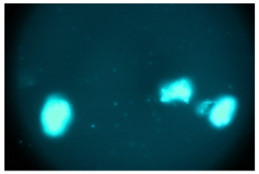<br>B. h. - IR<br>0 IgY<br>1/100 α ch - A568<br>(UV filter)	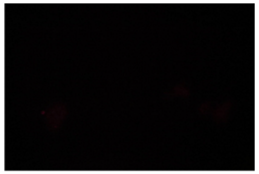<br>B. h. - IR<br>0 IgY<br>1/100 α ch - A568<br>(red filter)
